# Development of a ReaxFF Reactive Force Field for the Crystallization of van der Waals-Layered Bismuth Selenide

**DOI:** 10.1021/acs.jpcc.5c07042

**Published:** 2026-02-06

**Authors:** Ga-Un Jeong, Ryan Morelock, Soumendu Bagchi, Nadire Nayir, Adri C.T. van Duin, Panchapakesan Ganesh

**Affiliations:** a Department of Mechanical Engineering, The Pennsylvania State University, University Park, Pennsylvania 16802, United States; b Center for Nanophase Materials Sciences, 6146 Oak Ridge National Laboratory, Oak Ridge, Tennessee 37831, United States; c Paul-Drude-Institute for Solid State Electronics, Leibniz Institute within Forschungsverbund Berlin e.V., Hausvogteiplatz 5-7, Berlin 10117, Germany

## Abstract

Bismuth selenide (Bi_2_Se_3_) is a widely studied topological insulator and thermoelectric material whose properties are highly sensitive to crystal quality, defects, and stoichiometry. Recrystallization is an effective method of improving the crystal quality of materials, yet traditional experimental approaches are time-consuming and resource-intensive and often rely on trial and error. This work presents a new Bi/Se ReaxFF force field with the ability to recrystallize bulk Bi_2_Se_3_ into van der Waals (vdW)-layered phases under various thermal and kinetic conditions. The force field is parameterized using a diverse quantum mechanical data set, which includes formation energies of bulk layered and nonlayered Bi–Se phases, the energy–volume equation of state, point defect formation energies, the composition-dependent energetic stability trends of high-temperature Bi_*x*_Se_*y*_ clusters, and amorphous Bi_2_Se_3_ structures sampled from melt-quench molecular dynamics simulations. Our simulations reveal that structural characteristics of the resulting recrystallized vdW materials, such as stacking order and stoichiometry, depend on melt-quenching processing parameters such as the cooling rate and annealing temperature. This novel force field constitutes a predictive framework for the structural tuning of complex Bi–Se vdW materials through recrystallization conditions, laying a foundation for computational design of a much wider selection of chalcogenides.

## Graphical abstract

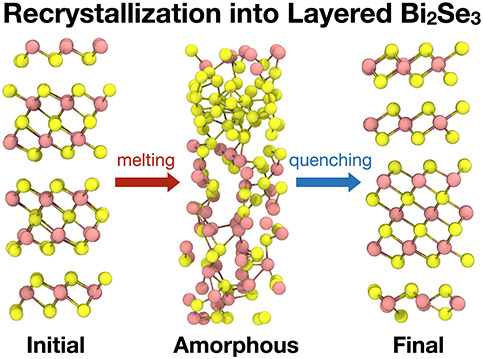

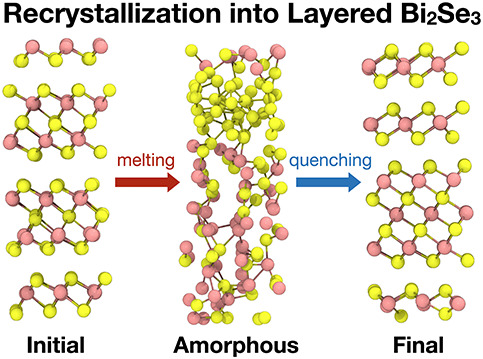

## 1 Introduction

Tetradymite-structured chalcogenides have garnered significant interest due to their exceptional properties as a topological insulators and thermoelectrics.^1,2^ Van der Waals-layered tetradymites with a crystal formula of A_2_X_3_, which have layers arranged as X­(I)-A-X­(II)-A-X­(I) (A = Bi or Sb; X = Se or Te; X­(I) and X­(II) denote inequivalent chalcogenide sites), have been shown to possess time-reversal symmetry-protected surface states that make them strong bulk topological insulators.^3^ Notably, 2D sheets of these materials, cleaved along the weakly bonded vdW interfaces, also exhibit topological insulating behavior with surface states that can be modified when in proximity to other surfaces.^4–6^ For example, the previous works suggest that Majorana states—exotic quasiparticles with potential applications in spintronics and fault-tolerant quantum computing—may emerge at the interface between 2D Bi_2_Se_3_ and conventional superconductors.^7–9^ Similarly, the low-dimensional atomic bonding results in significant higher-order anharmonic effects in A_2_X_3_ leading to its tunability for thermoelectrics.^10^

Realizing exotic quasiparticles or tunable anharmonicity in these 2D materials depends significantly on the heterojunction’s assembly, which can be broadly divided into mechanical stacking and bottom-up growth techniques.^11^ Mechanical stacking offers flexibility in layer arrangement by avoiding issues like lattice mismatch and in orientation by providing greater control over spatial properties like interlayer tilting.^12^ However, mechanical stacking sacrifices control over the interface, which can compromise emergent properties if defects or other irregularities occur in this sensitive region.^13^ In contrast, direct growth methods, such as molecular beam epitaxy (MBE), pulsed laser deposition (PLD), physical vapor deposition (PVD), or chemical vapor deposition (CVD), allow for greater interface quality control (e.g., crystallinity) but are highly sensitive to growth conditions, such as temperature, pressure, and chemical potential, as well as the choice of substrate, which can influence the nucleation and crystallinity of deposited films.^14^

Density functional theory (DFT) is a natural choice to explain structure–property relationships (e.g., interfacial and defect energies and epitaxial strain) in 2D topological heterojunctions near equilibrium, given that it describes chemical bonding with quantum mechanical accuracy.^15^ However, accurately expressing weak, long-range van der Waals interactions important for Bi_2_Se_3_ growth can be challenging for popular GGA functionals.^16^ Furthermore, modeling nonlocal defects like grain boundaries and low-angle twists between substrate and film layers can be complicated by periodic boundary conditions. Conventional DFT methods also cannot capture finite-temperature dynamics,^17^ and while ab initio molecular dynamics (AIMD)^18^ does, it is prohibitively expensive for time scales much longer than about 10 ps. Both methods are limited to systems with thousands of atoms, despite epitaxially grown Bi_2_Se_3_ having hundreds of angstroms of vdW layers.^19^

For these reasons, DFT and AIMD simulations of 2D materials are often complemented by molecular dynamics (MD) simulations^20^ that describe their time evolution classically, allowing for much longer time scales (e.g., picoseconds vs femtoseconds) and system sizes. Beyond traditional force fields^21^ that do not capture bond breaking or formation, reactive force fields including AIREBO^22^ and ReaxFF^23^ have been developed more recently to model energies and forces as continuous functions of interatomic distances. For example, ReaxFF uses a single parameter set per atom, irrespective of the charge state. This ensures physical consistency and reduces model complexity, which limits any additional expenses incurred compared to those of traditional force fields. Furthermore, ReaxFF force fields can be readily parameterized to DFT, AIMD, or even experimental data using a variety of optimizers developed by the community,^24–27^ meaning that it is adaptable and well-suited to model vdW-layered materials.^28,29^

In this work, we report a new, quantum mechanics (QM)-based ReaxFF force field, designed to simulate the recrystallization of amorphous Bi_2_Se_3_, a prominent member of tetradymite-structured chalcogenides, into van der Waals-layered phases using melt-quench molecular dynamics (MD) simulations. Our simulations revealed that the stacking sequence and stoichiometry of the recrystallized Bi_2_Se_3_ structure are influenced by the melt-quench parameters, such as the cooling rate, annealing temperature, and the MD duration, which determines the degree of atomic mobility during solidification. Therefore, this work represents an important step toward modeling the epitaxial synthesis of vdW-layered Bi–Se using ReaxFF reactive force fields. The remainder of this work is organized as follows: Section 2 provides an overview of the methods for this work. In Section 3, we present the force field parameterization and validate it via MD simulations recrystallizing amorphous Bi_2_Se_3_ under various melt-quenching parameters. The last section is devoted to our conclusion remarks.

## 2 Methodologies

### 2.1 ReaxFF Background

ReaxFF is a bond order-based reactive force field formalism that describes chemical reactions through dynamic bond formation and dissociation.^30^ The energy of the system is defined by eq 1:

$$E_{\mathrm{system}}=E_{\mathrm{bond}}+E_{\mathrm{over}}+E_{\mathrm{under}}+E_{\mathrm{lp}}+E_{\mathrm{val}}+E_{\mathrm{tor}}+E_{\mathrm{vdWaals}}+E_{\mathrm{Coulomb}}$$ 1

Bond order-dependent terms include contributions from *E*_bond_ (bond energy), *E*_over_ (overcoordination penalty energy), *E*_under_ (undercoordination penalty energy), *E*_lp_ (lone pair energy), *E*_val_ (valence angle energy), and *E*_tor_ (torsion angle energy). Bond orders are updated each MD step and are derived from local coordination environments, i.e., the distances to neighboring atoms. Nonbonded terms consist of *E*_vdWaals_ (van der Waals energy) and *E*_Coulomb_ (Coulomb energy) and are calculated for all atomic pairs. For this work, the atomic charges defining Coulomb interactions are calculated using the electronegativity equalization method (EEM),^31^ and van der Waals interactions are described with a Morse potential.^32^

### 2.2 Force Field Parameterization

#### 2.2.1 Quantum Mechanical Calculations

The ReaxFF training set for the Bi–Se system was constructed using periodic, projector-augmented wave (PAW)-based density functional theory (DFT) calculations as implemented in the Vienna ab initio simulation package (VASP) version 6.4.2.^33–35^ We chose the generalized gradient approximation (GGA) Perdew–Burke–Ernzerhof (PBE) exchange–correlation (XC) functional^36^ and included long-range van der Waals interactions using the Grimme DFT-D3 method with Becke–Johnson damping. The electronic wave function basis set was expanded with an energy cutoff of 520 eV, and Gaussian smearing was used for partial occupancies with a smearing width of 0.03 eV. Spin-polarized electronic structure calculations were employed for calculating total energies. Gamma-centered *k*-point meshes with grid densities of ≥2000/number of atoms were automatically generated for all structures using the Pymatgen Python package.^37^ Bulk structures queried from Materials Project and included as training data were ionically and cell-relaxed with an energy convergence criterion of 0.0001 eV/atom and a force convergence criterion of 0.01 eV/Å. For all other structures included as training data, only single-point calculations were performed, with an energy convergence criterion of 0.0001 eV/atom. Atomic charges were obtained using Bader analysis, which assigns charges to atoms based on zero-flux surfaces in the charge density computed with VASP.

We calculated the equation of states (EoS) for rhombohedral Bi_2_Se_3_, rocksalt BiSe, and van der Waals-layered Bi_8_Se_9_ by varying the volume from 85% to 115% of the optimal volume. Although rocksalt BiSe and layered Bi_8_Se_9_ lie slightly above the 0 K convex hull (i.e., are metastable), including their EoS’s allows us to capture the influence of bond distances on energetics for systems with coordination environments beyond vdW-layered Bi_2_Se_3_. We expect to improve our force field’s lattice constant predictions and behavior under variable volumes (e.g., the NPT ensemble) by fitting to these off-equilibrium structures. We also included point defect formation energies for the vdW Bi_2_Se_3_ structure in our training data, using the pymatgen-analysis-defects package:^37^ antisite (e.g., Bi → Se), interstitial (within vdW layers and interlayer spaces), and vacancies (∼11% Se defect concentration) (Figure S1). In the calculations, atomic positions of the defective structures were relaxed, while the lattice parameters were kept fixed.

#### 2.2.2 Parameterization

Our force field training data include the DFT energies of periodic crystalline structures with energy–volume equations of states (Bi_2_Se_3_, BiSe, and Bi_8_Se_9_) and point defects (Bi_2_Se_3_), periodic amorphous structures, and nonperiodic Bi_*x*_Se_1–*x*_ clusters with varying stoichiometries. Starting from the previously published Bi^38^ and Se^39^ force fields, we optimized the Bi–Se bond and off-diagonal and Bi–Se–Bi, Se–Bi–Se, Se–Se–Bi, and Bi–Bi–Se valence angle parameters using a successive single-parameter parabolic extrapolation approach.^40^ The newly developed parameter set is presented in the SI. More comprehensive descriptions of the ReaxFF framework and procedures to optimize its parameters can be found in our earlier publications.^41,42^

### 2.3 MD Simulations

We investigated recrystallization behavior with melt-quench molecular dynamics simulation run with the Amsterdam Density Functional (ADF) software^43^ and LAMMPS.^44^ A 135-atom periodic supercell of trigonal, vdW-layered Bi_2_Se_3_ was equilibrated at 300 K for 25 ps in an NPT ensemble with a time step of 0.25 fs using a Berendsen thermostat and barostat with temperature and pressure damping constants of 100 and 5000 fs, respectively, to minimize the artificial stress and strain, thus bringing the system to a local potential energy minimum. The density following equilibration was 6.83 g/cm^3^, corresponding to a box size of 12.73 × 12.69 × 30.73 Å. Subsequent melt-quench simulations were performed using an NVT ensemble with a time step of 0.5 fs. The system was heated from 0 to 5000 K at a rate of 20 K/ps, held at 5000 K for 50 ps, and then cooled to 2000 K at a rate of 2 K/ps. After maintaining the temperature at 2000 K for 100 ps, the system was quenched to 0 K at the same rate. These temperature profiles were chosen to ensure sufficient melting and controlled recrystallization during cooling, consistent with a previous study on similar vdW systems.^28^ We used partial radial distribution functions to evaluate the quality of recrystallized Bi_2_Se_3_.

## 3 Results and Discussion

### 3.1 ReaxFF Reactive Force Field Parameterization

#### 3.1.1 Crystalline Structures

The DFT convex hull captures heats of formation of competing phases and thus describes their relative energetic stability at 0 K. An accurate and performant force field for Bi–Se recrystallization should reproduce, or closely trend with, the relative stabilities of experimentally observed phases. For Bi–Se binary systems, the heat of formation (Δ*H*_f_) is given by eq 2:

$$\Delta H_{f}=E_{{\mathrm{Bi}}_{x}{\mathrm{Se}}_{1-x}}-xE_{\mathrm{Bi}}-(1-x)E_{\mathrm{Se}}$$ 2

where *x* is the Bi atomic fraction in Bi_*x*_Se_1–*x*_, $E_{{\mathrm{Bi}}_{x}{\mathrm{Se}}_{1-x}}$ is the total energy per atom for the Bi_*x*_Se_1–*x*_ phases, and *E*_Bi_ and *E*_Se_ are the per-atom reference energies of the bulk rhombohedral Bi and trigonal Se, respectively. We selected 11 crystalline structures in the Bi_*x*_Se_1–*x*_ compositional space from the Materials Project database,^45^ including the vdW-layered Bi_2_Se_3_ (trigonal, *R*3̅*m*) and layered phases containing Bi_2_ interlayers, such as BiSe (trigonal, *P*3̅*m*1), Bi_4_Se_3_ (trigonal, *R*3̅*m*), and Bi_8_Se_9_ (trigonal, *R*3̅*m*). There are many possible combinations of stacked Bi_2_ and Bi_2_Se_3_ layers in the Bi–Se compositional system: as described by Okamoto,^46^ compounds with any concentration can be configured in the composition range between 0 and 60 at. % Se by stacking these layers. Of the 11 crystalline structures used to construct the convex hull, only Bi_4_Se_3_, BiSe, and Bi_8_Se_9_ are Bi_2_-based stacked layered phases, which have ∼43 atom % Se, 50 atom % Se, and ∼53 atom % Se, respectively. These structures represent a diverse range of compositions with varying stoichiometry ratios and symmetries, with DFT-predicted stabilities spanning from the convex hull (e.g., Bi_2_Se_3_ (trigonal, *R*3̅*m*)) to 330 meV/atom above it (e.g., BiSe (tetragonal, *I*4/*mmm*)). Figure 1 compares the formation energies computed by DFT to those predicted by our ReaxFF force field. Figure 1a shows that Bi_2_Se_3_ (trigonal, *R*3̅*m*) is predicted by ReaxFF to be the most stable phase, in agreement with DFT. Figure 1b shows the same set of stable and metastable phases included in the convex hull and confirms that all competing phases above the DFT convex hull are also predicted to be metastable by ReaxFF. These results indicate that the developed force field captures the overall energy trends of the convex hull. While our force field overstabilizes Bi_2_Se_3_ by approximately 4.59 kcal/(mol atom) (ReaxFF −13.73 kcal/(mol atom), DFT −9.14 kcal/(mol atom)), this deviation is considered acceptable, as a similar magnitude of overstabilization (∼3 kcal/(mol atom)) was previously reported by Ponomarev et al. for a force field that successfully recrystallized vdW-layered MoS_2_.^28^ This behavior can be attributed to the observation that recrystallization simulations tend to yield more consistent results when the heat of formation of Bi_2_Se_3_ (trigonal, *R*3̅*m*) is at least 2 kcal/mol lower than that of competing phases, and this trend was considered during the parameterization.

**Figure 1:**
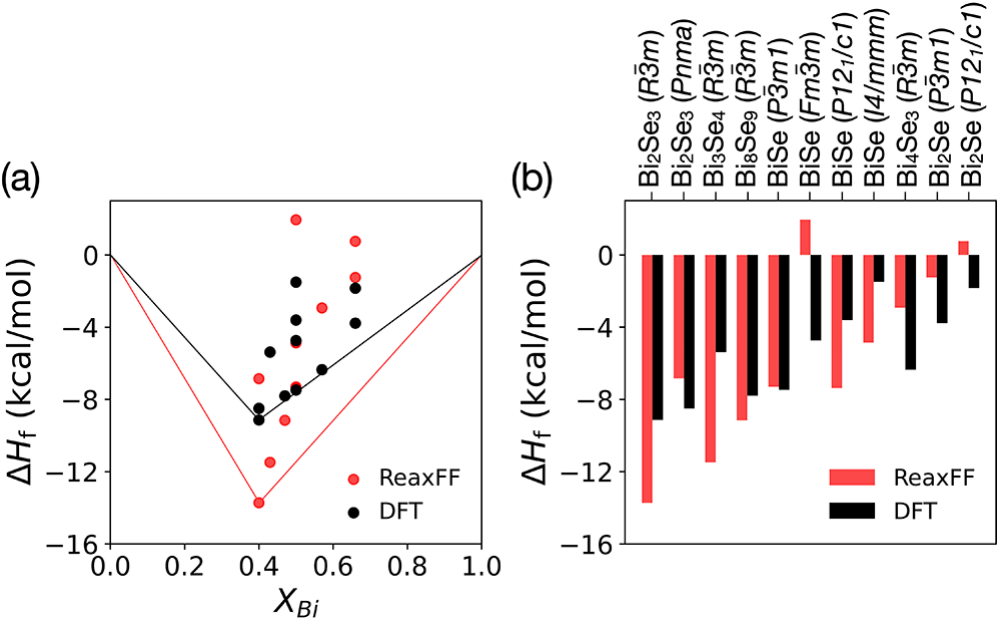
(a) Convex hull as a function of Bi fraction in Bi_*x*_Se_1–*x*_, depicted by *X*_Bi_, and (b) bar chart comparing the per-atom formation energies (Δ*H*_f_) of bulk Bi_*x*_Se_1–*x*_ compounds computed with DFT (black) and predicted by our new ReaxFF force field (red).

Figure 2 provides additional support to Figure 1, showing that ReaxFF reproduces the DFT energy–volume curves near equilibrium for all three phases and captures EoS trends, even far from equilibrium. For Bi_2_Se_3_, however, the force field overestimates the energetic penalties at extreme volumes (<94% or >103% of the equilibrium volume). In addition, we confirmed that ReaxFF reproduces the atomic charges of Bi_2_Se_3_ and BiSe structures in good agreement with DFT (see Table S1 in the Supporting Information).

**Figure 2:**
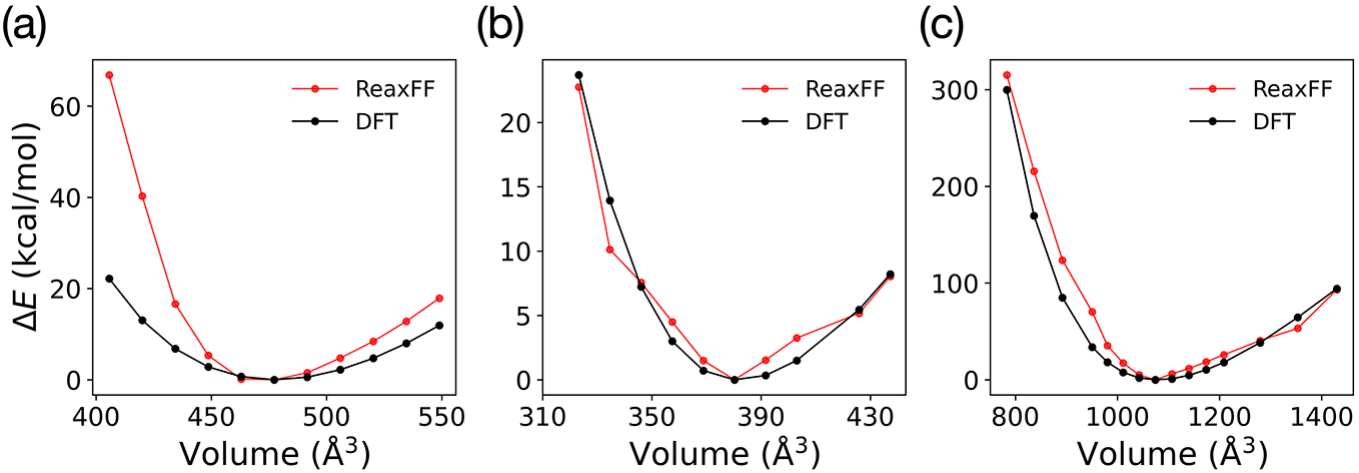
Equations of states as a function of the rescaled lattice parameter for experimentally observed compounds (a) vdW-layered Bi_2_Se_3_ and (b) rocksalt BiSe and (c) Bi_8_Se_9_, with Bi_2_ interlayers between stacked Bi_2_Se_3_ layers.

Amorphization is often mediated by the formation of defects (antisite, interstitial, or vacancy), and we expect accurate defect energies to be a strong indicator of a force field’s ability to recrystallize. Our training data included the formation energies of vdW-layered Bi_2_Se_3_ with a single vacancy (removed atom) defect, five types of interstitial (added atom) defects, and three types of substitutional (swapped atom) defects. We defined the formation energies (*E*_f_(*D*)) of the point defect models by eq 3:

$$E_{f}(D)=E_{\mathrm{defect}}-E_{\mathrm{pristine}}-\sum_{i}n_{i}\mu_{i}$$ 3

where *E*_defect_ and *E*_pristine_ are the total energies of the defective and pristine bismuth selenide (Bi_2_Se_3_) system, respectively. *n*_*i*_ denotes the number of atoms of type *i* (Bi or Se) added (*n*_*i*_ > 0) or removed (*n*_*i*_ < 0) to form defects, and μ_*i*_ is the chemical potential of atom *i*, referenced to the energies of Bi and Se atoms in the bulk forms of rhombohedral Bi and trigonal Se, respectively. We modeled point defects in vdW-layered Bi_2_Se_3_ that are relevant to recrystallization. Defects were grouped into three classes: (i) vacancies, represented by a single Se vacancy (V_Se_); (ii) interstitials, Bi_i_ and Se_i_, located either in the van der Waals (vdW) gap between adjacent quintuple layers (QLs, Se–Bi–Se–Bi–Se) or within a single QL; and (iii) antisites, Bi_Se_ (Bi occupying a Se lattice site) and Se_Bi_ (Se on a Bi site). Detailed structural models are provided in Figure S1.

Figure 3 compares defect energy predictions made by our ReaxFF force field to DFT. ReaxFF tends to overestimate the defect formation energies of Bi_2_Se_3_, which is consistent with the overstabilization of pristine Bi_2_Se_3_ relative to Bi and Se references (Figure 1) and with the overpenalization at extreme volumes observed in the energy–volume curve (Figure 2a). The largest discrepancies occur for defects within or at the boundaries of the vdW gap, specifically interstitial Bi_i_ and Se_i_ in the vdW gap between adjacent QLs and Se_Bi_ antisite defects located at a vdW gap boundary, low-coordination environments that are highly sensitive to changes in the local volume and coordination. By contrast, defects within a QL are relatively well-captured. Overall, ReaxFF predicts endothermic formation energies for all point defects in Bi_2_Se_3_ considered, in qualitative agreement with DFT.

**Figure 3:**
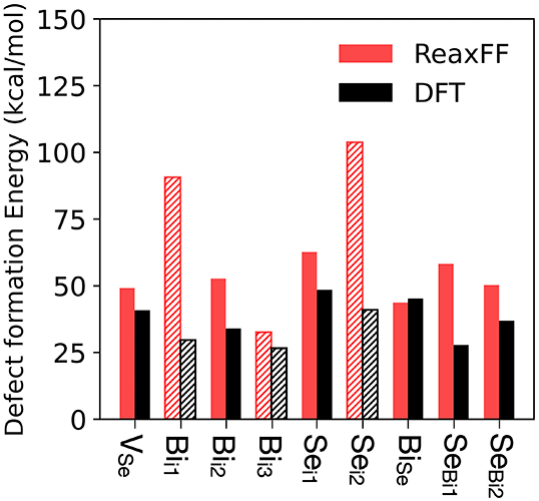
Defect configurations in crystalline Bi_2_Se_3_ and their formation energies from ReaxFF (red) compared with DFT (black): Se atom vacancy (V_Se_), Bi atom interstitial (Bi_i_), Se atom interstitial (Se_i_), and antisites in which the Bi atom occupies a Se site (Bi_Se_) and the Se atom occupies a Bi site (Se_Bi_). For interstitial defects, bars corresponding to sites within a quintuple layer are shown with solid fill, whereas those in the vdW gap are hatched.

#### 3.1.2 Disordered and Nonequilibrium Structures

Periodic amorphous Bi_2_Se_3_ structures were added to improve the force field’s accuracy during melt-quench cycling, following the methodology of Ponomarev et al. for MoS_2_.^28^ In a closed-loop workflow, we generated 40 amorphous Bi_2_Se_3_ structures by MD melt-quench simulations from 5000 to 0 K at 20 K/ps. We computed the single-point energies of these images and normalized them with respect to the crystalline Bi_2_Se_3_ phase, after which they were added to the training set and used to retrain the force field. As shown in Figure 4, there is a strong correlation (*R* ^2^ = 0.946) between the energies predicted by our ReaxFF force field and the energies computed with DFT (fitted slope close to 1.057).

**Figure 4:**
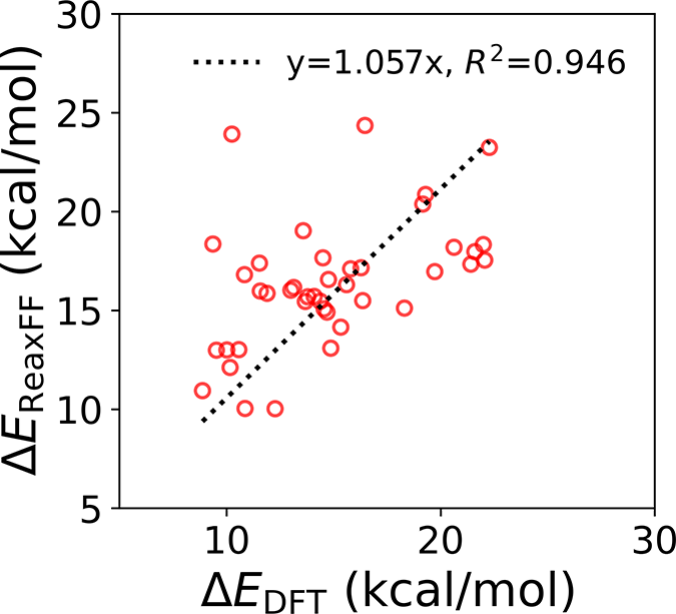
Relative per-atom energies of amorphous Bi_2_Se_3_ structures, defined as Δ*E* = *E*_amorphous_ – *E*_crystal_, computed with DFT and ReaxFF. The dotted line represents a linear regression fit forced through the origin (slope = 1.057, *R* ^2^ = 0.946), indicating that ReaxFF captures the DFT-predicted energy scale and relative trends across the amorphous configurations.

While fitting our ReaxFF model to Bi–Se crystalline phases can allow it to reproduce certain material properties, fitting exclusively to bulk structures, even amorphous bulk structures, does not guarantee that melt dynamics will be accurately captured. To improve our Bi/Se ReaxFF force field so that it better captures liquid-to-solid transition behavior, we also included a diverse set of Bi–Se cluster configurations in our training data, which were generated via high-temperature MD simulations. We embedded a Bi_12_ cluster into an environment containing 30 Se atoms at a temperature of 800 K, which is above the melting point but below the evaporation point of Bi. To control the thermal behavior, two separate thermostats were applied: a strong thermostat (10^3^ fs) maintained the temperature of Bi atoms to around 800 K, while a very weak thermostat (10^8^ fs) was applied to Se atoms to suppress Se–Se clustering. During the simulation, reactions between the Bi cluster and surrounding Se atoms led to the formation of Bi_*x*_Se_*y*_ clusters with various compositions. Single-point DFT calculations were performed on 40 representative Bi–Se clusters from the MD trajectory. Figure 5 shows the relative energies of 40 Bi_*x*_Se_*y*_ clusters, generated from high-temperature MD simulations, as computed by both ReaxFF and DFT. Although the relative energies tend to be underestimated, the force field consistently predicts all Bi–Se clusters across various compositions to be less stable than the Bi_2_Se_3_ crystal. Given that there is always a small difference between the potential energy surface (PES) learned by ReaxFF and the underlying Born–Oppenheimer surface described by DFT, it is expected that for structures extracted from ReaxFF-based MD simulations (as in Figure 5), the ReaxFF energies will be systematically lower than their DFT equivalents.

**Figure 5:**
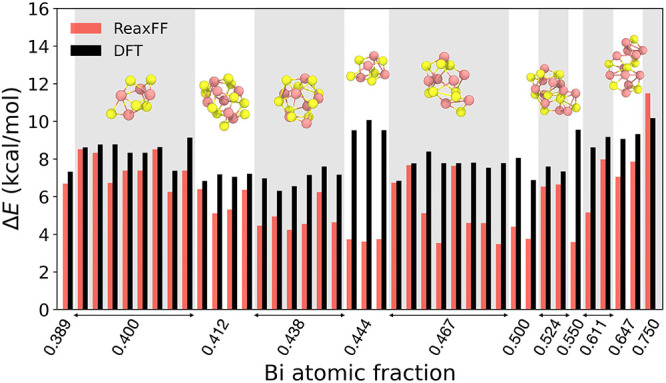
Relative per-atom energies of 40 Bi_*x*_Se_*y*_ clusters extracted from high-temperature MD simulations, with respect to the Bi_2_Se_3_ crystal structure, as computed by both ReaxFF and DFT. Bi: light red; Se: yellow.

### 3.2 Recrystallization of Amorphous Bi_2_Se_3_: Molecular Dynamics Simulations

We evaluated our force field’s ability to recrystallize Bi_2_Se_3_ using the melt-quench protocol outlined in Section 2.3 and the Amsterdam Density Functional (ADF) software. Final structures and radial distribution functions from these simulations are shown in Figure 6 (LAMMPS simulation results are provided in Figure S2). The vdW-layered bulk Bi_2_Se_3_ structure was first equilibrated at 300 K under the NPT ensemble (Figure 6a,d) before being rapidly amorphized during a 250 ps temperature ramp from 0 to 5000 K (ramp rate of 20 K/ps). Following annealing at 5000 K for 50 ps (Figure 6b,e), the structure was cooled to 0 K at a rate of 2 K/ps (Figure 6c,f).

**Figure 6:**
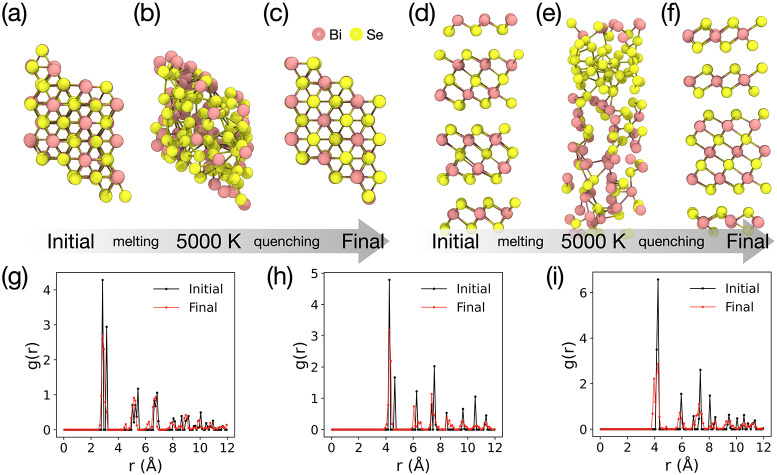
(a) Snapshots of 135-atom Bi_2_Se_3_ structures along the MD trajectory of our melt-quench recrystallization simulations. Top and side views (a, d) of the initial vdW-layered crystalline structure, equilibrated at 300 K via an NPT ensemble, (b, e) amorphous structure following temperature ramping to 5000 K, and (c, f) final structure following quenching to 0 K. Radial distribution functions of the final structure generated via melt-quenching for (g) Bi–Se, (h) Bi–Bi, and (i) Se–Se.

Figure 6g–i compares the partial radial distribution functions (RDFs) of Bi–Se, Bi–Bi, and Se–Se interatomic distances in the final, recrystallized structure to those in the initial crystalline structure. The first-neighbor peak of the Bi–Se bond in the quenched structure closely matches that of the pristine crystal (Figure 6g), indicating that the short Bi–Se bond lengths are recovered. The characteristic splitting of the first Bi–Se peak in the crystal, which arises from two inequivalent Bi–Se distances in the BiSe_6_ octahedra, is not fully captured after quenching and collapses into a single broadened peak. In addition to the nearest-neighbor peaks, longer-range peaks are also reasonably well-reproduced in terms of their positions and general shapes, although they are slightly broadened. This shows that the structural order of the pristine crystal is generally recaptured during quenching. Similarly, the Bi–Bi and Se–Se pair distribution functions (Figure 6h,i) of the quenched structure exhibit reasonable agreement with those of the pristine structure, not only for the nearest neighbors but also for the longer range.

The structure at 5000 K shows phase separation into a Se-rich phase. This behavior can be explained by the heat of formation diagram presented in Figure 1: energetically stable Se-rich Bi_*x*_Se_1–*x*_ phases tend to form at relatively moderate temperatures. Higher temperatures typically favor the formation of the Bi-rich Bi_*x*_Se_1–*x*_ phases that occupy the upper region of the diagram, resulting in the segregation of Se in the remaining regions. Visual inspection of the recrystallized bulk structure reveals four vdW layers: three resembling layered BiSe_2_ and one like Bi_3_Se_4_. The Bi_3_Se_4_ (trigonal, *R*3̅*m*) observed in the final configuration has been reported experimentally and is also described to be thermodynamically highly favorable by our force field, with a formation energy of −11.49 kcal/(mol atom), about 2.24 kcal/(mol atom) less stable than Bi_2_Se_3_ (trigonal, *R*3̅*m*)). Consequently, the system appears to have converged to a local minimum by following a kinetically accessible pathway rather than the global thermodynamic minimum (Bi_2_Se_3_). Consistently, the final structure exhibits an energy approximately 106.56 kcal/mol higher than that of the initial configuration.

During this melt-quench simulation, the Bi_3_Se_4_ layer forms first, followed by the organization of the remaining atoms into BiSe_2_ layers depending on the local chemical environment (Figure S3b). Notably, the first layer formation is driven by an excess chalcogen chemical potential, which aligns with the experimental observation from the MBE growth of Bi_2_Se_3_ thin films, where a Se-rich atmosphere is known to control the formation of stoichiometry and quality of the films.^47–49^ Under the small-cell conditions and melt-quench protocol employed herein, the force field reproduces key indicators of vdW layer formation and achieves recrystallization but not with exact stoichiometry and stacking of the ground-state crystal. Instead, the final structure remains metastable and higher in energy than the ideal layered crystal. Nevertheless, we discover a critical kinetic control of stacking in vdW crystals formed by recrystallization: choosing a temperature that favors different degrees of Se-rich phase formation allows control over the composition of the initial Bi-rich layer and thereby the type of stacking we will achieve in the final recrystallized material, enabling control over its phase.

To further explore this kinetic control of vdW stacking, we performed recrystallization simulations at different maximum melting temperatures (4000, 5000, 6000, and 7000 K) and compared the final structures. Apart from variations in melting temperature, the MD protocol follows the same procedure described in Section 2.3. As shown in Figure 7, the number and type of van der Waals layers formed change depending on the maximum melting temperature. At 4000 and 5000 K, three layers resembling BiSe_2_ and one layer resembling Bi_3_Se_4_ are observed, whereas at 6000 K, two layers resembling BiSe_2_ and one layer resembling Bi_4_Se_5_ are formed, and at 7000 K, a single layer resembling Bi_8_Se_9_ is formed. As the maximum melting temperature increases, the system tends to form more Bi-rich phases (Bi_2_Se_3_: 40% Bi, Bi_3_Se_4_: 43% Bi, Bi_4_Se_5_: 44% Bi, and Bi_8_Se_9_: 47% Bi). This suggests that the composition of the resulting van der Waals layers can be tuned by controlling the temperature.

**Figure 7:**
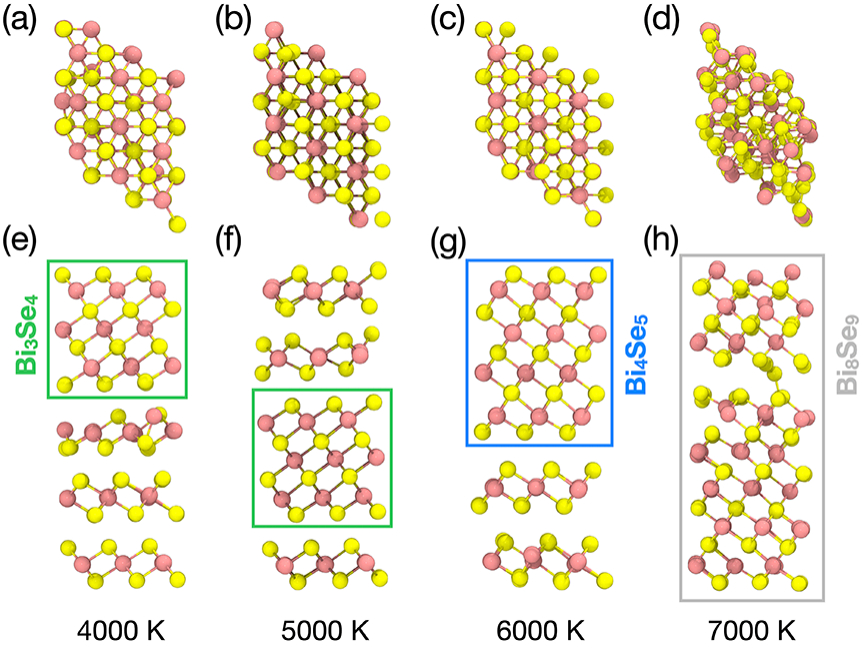
Recrystallization as a function of the maximum melting temperature. Top and side views after melt-quench cycles with *T*_max_ of (a, e) 4000 K, (b, f) 5000 K, (c, g) 6000 K, and (d, h) 7000 K. In the side views, the green, blue, and gray boxes mark vdW layer compositions of Bi_3_Se_4_, Bi_4_Se_5_, and Bi_8_Se_9_, respectively. Unboxed layers correspond to BiSe_2_.

We performed larger-scale melt-quench molecular dynamics (MD) simulations for a 3645-atom Bi_2_Se_3_ system, using the melt-quench protocol described in Section 2.3, to assess the transferability of our force field beyond 135-atom systems. Figure 8 shows the structural evolution through the melt-quench process. At 5000 K (Figure 8a), the favoring of Bi-rich Bi_*x*_Se_1–*x*_ phases at high temperatures leads to the segregation of Se in the remaining regions, consistent with the behavior observed in the small-scale simulations. At 3600 K during quenching (Figure 8b), the Se-segregated region becomes more pronounced; the remaining regions show slight Bi enrichment and remain uniformly mixed without observable layering. Upon further quenching to 1700 K (Figure 8c), the Se-segregated region contracts, and Bi–Se bonds preferentially form near the Se-rich regions, indicating the early growth of slightly Se-enriched Bi–Se layered structures. This behavior results from the increased stability of Se-rich Bi_*x*_Se_1–*x*_ phases at lower temperatures. Se atoms in the segregated region feed into the surrounding melt, establishing local Bi–Se order near that region, and a Bi-rich residual region forms elsewhere (lower right). In the fully quenched state (Figure 8d), we observe the Se layers, Bi–Se layered regions with multiple orientations, and a Bi-rich disordered region. Most layered structures are close to BiSe_2_ composition (33% Bi), whereas the broad mixed region at the bottom is near equiatomic composition (50% Bi, *z* ≈ 35 Å). The observed compositions are consistent with convergence toward the convex-hull minimum at Bi_2_Se_3_, but quenching inhibits sufficient ordering, and the system resides in a local metastable minimum. Comparing the Se-rich and Bi-rich regions, the higher mobility of Se enables two-dimensional ordering, whereas the heavier Bi atoms seem to rearrange very slowly and are quenched into a disordered state. We expect that longer MD simulation times with slower quench rates are necessary to fully recrystallize the pristine Bi_2_Se_3_ phase or other layered stackings of the Bi-rich and Se-rich crystalline phases. Infinitely slow quench rates should recover the pristine Bi_2_Se_3_ phase, but it is beyond the scope of this investigation. Nevertheless, the observed Se segregation suggests that variable temperature annealing can be used to control stacking in the quenched crystalline material, thereby controlling its phase.

**Figure 8:**
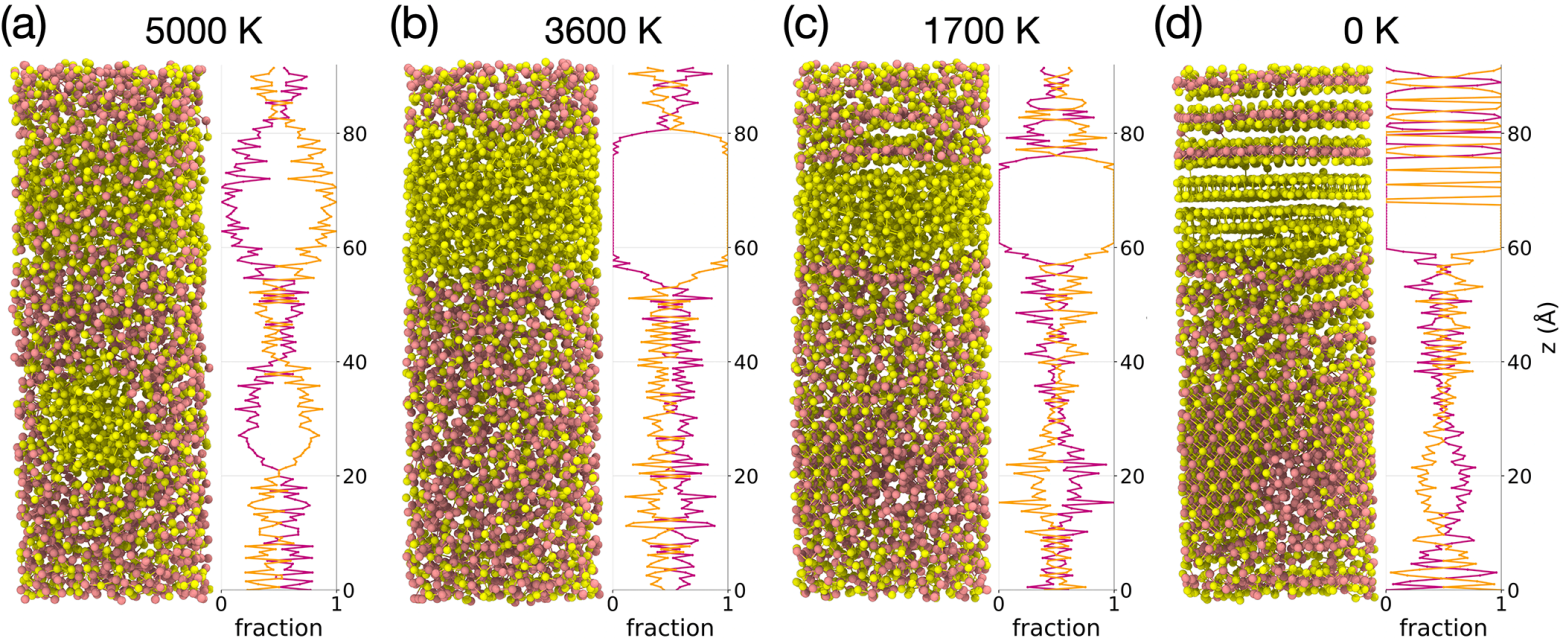
Side-view snapshots (left) and composition profiles (right) of a 3645-atom Bi_2_Se_3_ system along a melt-quench MD trajectory. (a) Structure after ramping to 5000 K (end of melting) and structures during quenching at (b) 3600 K, (c) 1700 K, and (d) 0 K. Composition profiles report the atomic fractions of Bi (magenta) and Se (yellow) as a function of the *z* coordinate.

## 4 Conclusions

We developed a ReaxFF reactive force field for the Bi–Se system that models the recrystallization of Bi_2_Se_3_ into van der Waals-layered phases under high-temperature melt-quench conditions. The force field was trained with a comprehensive DFT data set that included stable and metastable phases, defect configurations, amorphous states, and high-temperature Bi–Se clusters. Melt-quench simulations confirmed that the Bi/Se ReaxFF force field can reconstruct vdW-layered Bi_2_Se_3_ structures from the amorphous states. The recrystallized structures have a range of layered motifs, including BiSe_2_, Bi_3_Se_4_, and Bi_4_Se_5_-like layers, with their composition and arrangement varying depending on the maximum melting temperature. As the melting temperature increased, the formation of Bi-rich layers became more dominant, indicating that temperature-driven phase selection follows thermodynamic stability, and in conjunction with the rate of quenching can give kinetic control over the recrystallized phase with targeted vdW stacking. These results demonstrate that the force field captures the thermal sensitivity of the vdW layer composition and structure, suggesting that recrystallized Bi–Se phases can be tuned via thermal processing, providing a guiding principle to different experimental protocols for synthesizing controlled vdW homo- and heteroepitaxial 2D materials. Overall, the developed Bi/Se ReaxFF force field enables atomistic modeling of recrystallization and vdW layer formation with composition and temperature-dependent sensitivity.

## Supplementary Material



## Notes

Comparison of atomic charges obtained from ReaxFF and DFT (Table S1), defect configurations in crystalline Bi_2_Se_3_ (Figure S1), melt-quenching simulation using LAMMPS (Figure S2), melt-quench recrystallization trajectories showing the time evolution of vdW layer formation (Figure S3), and ReaxFF reactive force field parameters for Bi/Se (PDF)
