# Supplementary material for: Development of a ReaxFF Reactive Force Field for the Crystallization of van der Waals-Layered Bismuth Selenide
Source: J Phys Chem C Nanomater Interfaces. 2026 Feb 6;130(7):2744–52. doi: 10.1021/acs.jpcc.5c07042 (PMC12927021; doi:10.1021/acs.jpcc.5c07042)
Supplement: Supplementary file 1 [file jp5c07042_si_001.pdf]

SUPPORTING INFORMATION

Development of a ReaxFF Reactive Force Field for the crystallization of van der Waals layered Bismuth Selenide

Ga-Un Jeong[*a], Ryan Morelock[*b], Soumendu Bagchi[*b], Nadire Nayir[c], Adri C.T. van Duin[*a], Panchapakesan Ganesh[*b]

[a] *Department of Mechanical Engineering, The Pennsylvania State University, University Park, PA, 16802, USA*

[b] *Center for Nanophase Materials Sciences, Oak Ridge National Laboratory, Oak Ridge, TN 37831, USA*

[c] *Paul-Drude-Institute for Solid State Electronics, Leibniz Institute within Forschungsverbund Berlin eV., Hausvogteiplatz 5-7, 10117 Berlin, Germany*

[*] Email (corresponding authors): Adri C.T. van Duin (acv@psu.edu), Panchapakesan Ganesh (ganeshp@ornl.gov), Ga-Un Jeong (gjj5162@psu.edu), Ryan Morelock (morelockrj@ornl.gov), and Soumendu Bagchi (bagchis@ornl.gov)

Table S1. Comparison of atomic charges obtained from ReaxFF and DFT for vdW-layered Bi_2Se_3 and rocksalt BiSe crystal structure.

		ReaxFF	DFT
Bi_2Se_3 ($R\bar{3}m$)	Bi	0.8145	1.0005
	Se (within QL)	-0.8113	-0.8209
	Se (at vdW gap)	-0.4089	-0.5876
BiSe ($P\bar{3}m1$)	Bi	0.7802	0.9830
	Se	-0.6685	-0.6557

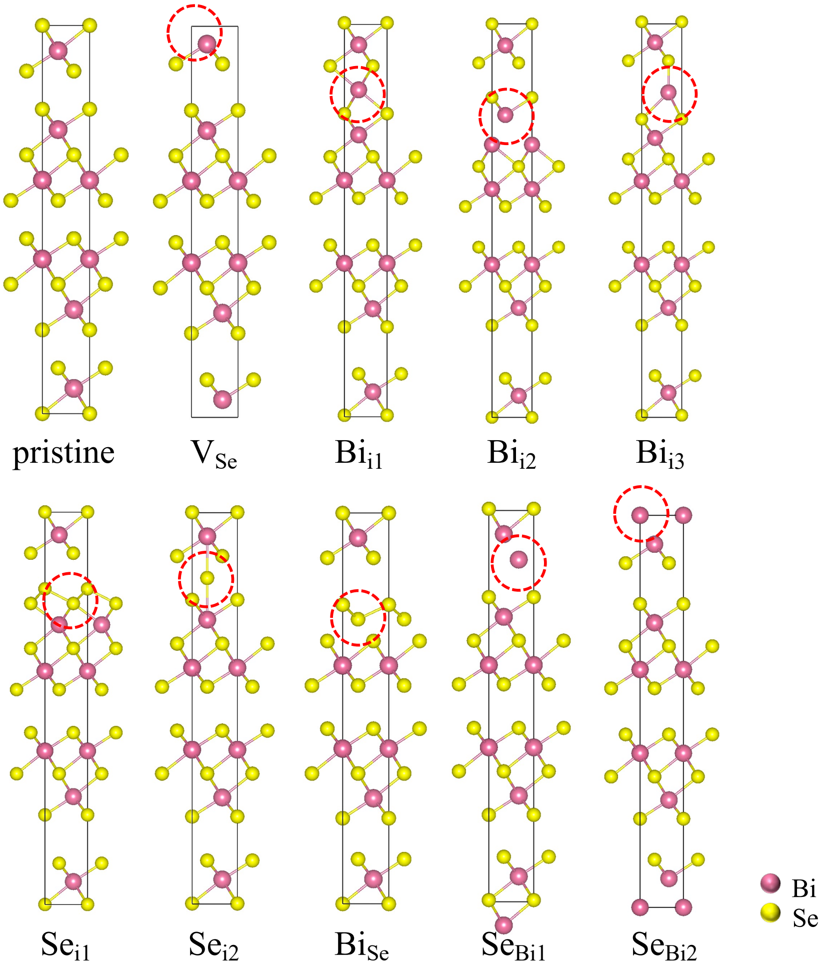

Figure S1. Defect configurations in crystalline Bi_2Se_3: Se atom vacancy (V_{Se}), Bi atom interstitial (Bi_i), Se atom interstitial (Se_i), and antisites in which the Bi atom occupies a Se site (Bi_{Se}), and the Se atom occupies a Bi site (Se_{Bi}).

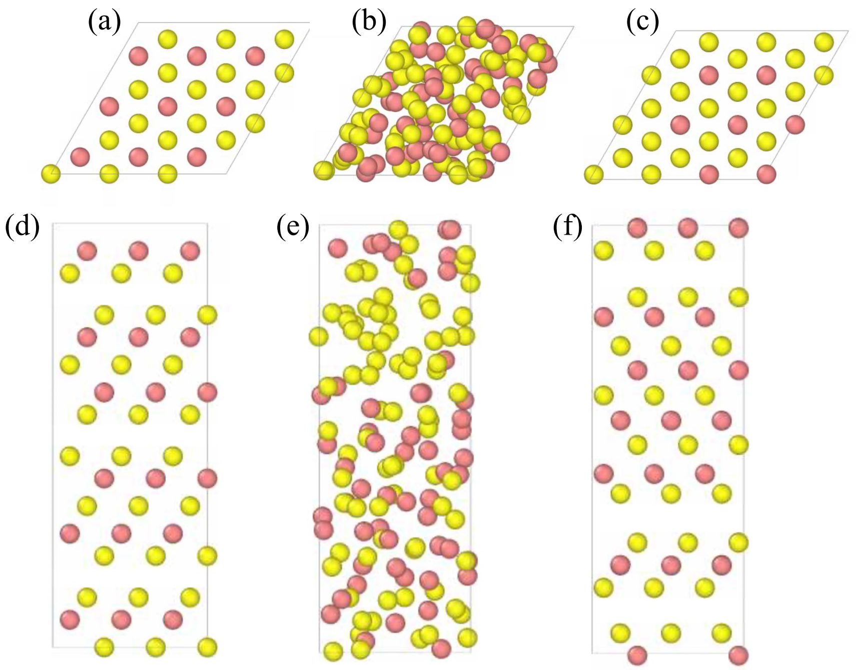

Figure S2. Structures representing the melt-quenching process obtained from LAMMPS simulations using the developed ReaxFF force field with the Nose–Hoover thermostat. Top and side views of (a,d) the initial structure, (b,e) structure following melting up to 5000 K, and (c,f) final structure after quenching to 0 K.

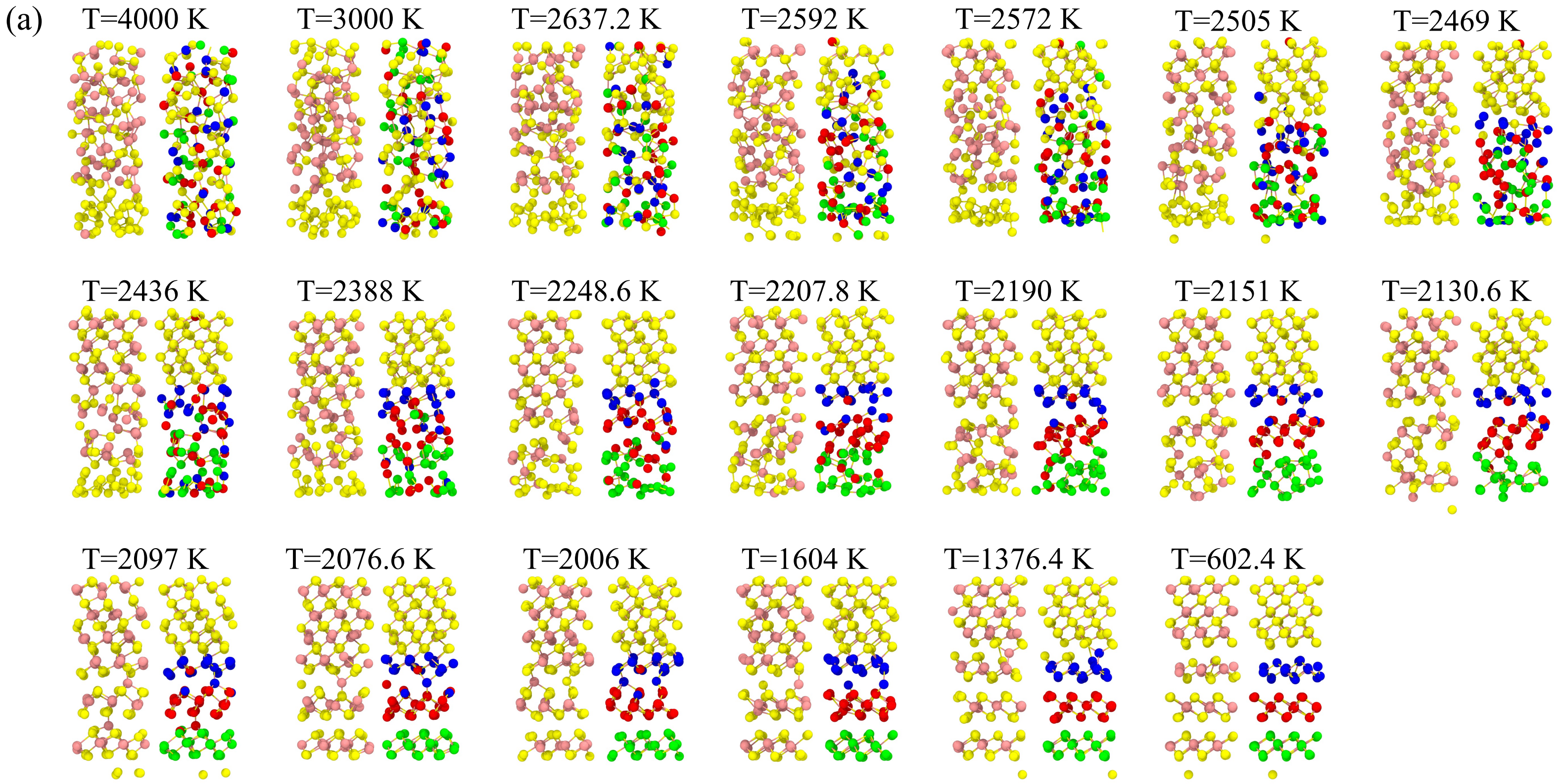

Figure S3. Snapshots of the melt-quench recrystallization trajectories at (a) 6000 K, (b) 5000 K, and (c) 4000 K, showing the time evolution of van der Waals (vdW) layer formation. In each snapshot, the left panel shows atomic configurations colored by element (Bi: light red, Se: yellow), while the right panel highlights atoms based on their vdW layer assignment, with different colors indicating different layers.

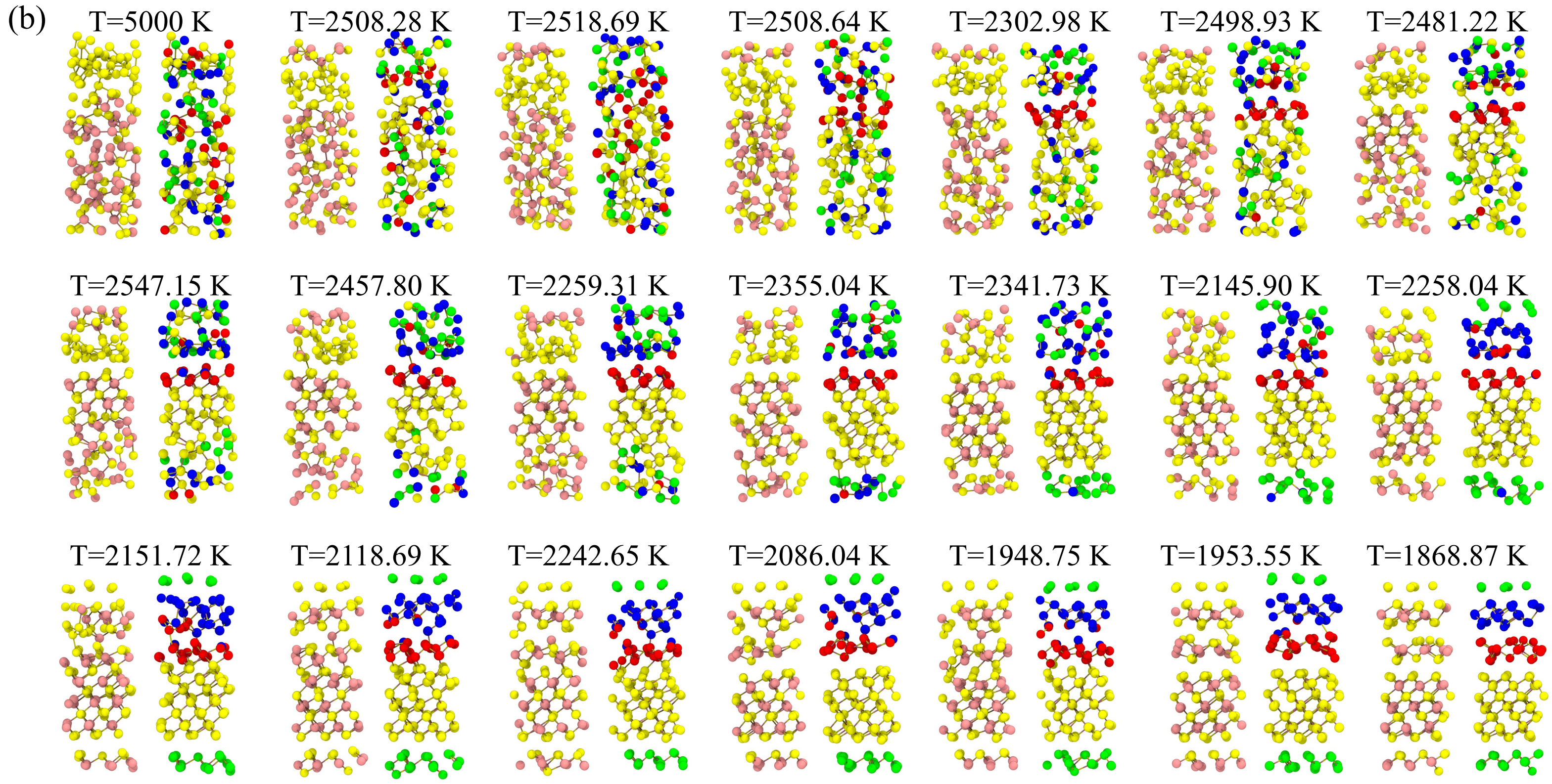

Figure S3. Snapshots of the melt-quench recrystallization trajectories at (a) 6000 K, (b) 5000 K, and (c) 4000 K, showing the time evolution of van der Waals (vdW) layer formation. In each snapshot, the left panel shows atomic configurations colored by element (Bi: light red, Se: yellow), while the right panel highlights atoms based on their vdW layer assignment, with different colors indicating different layers.

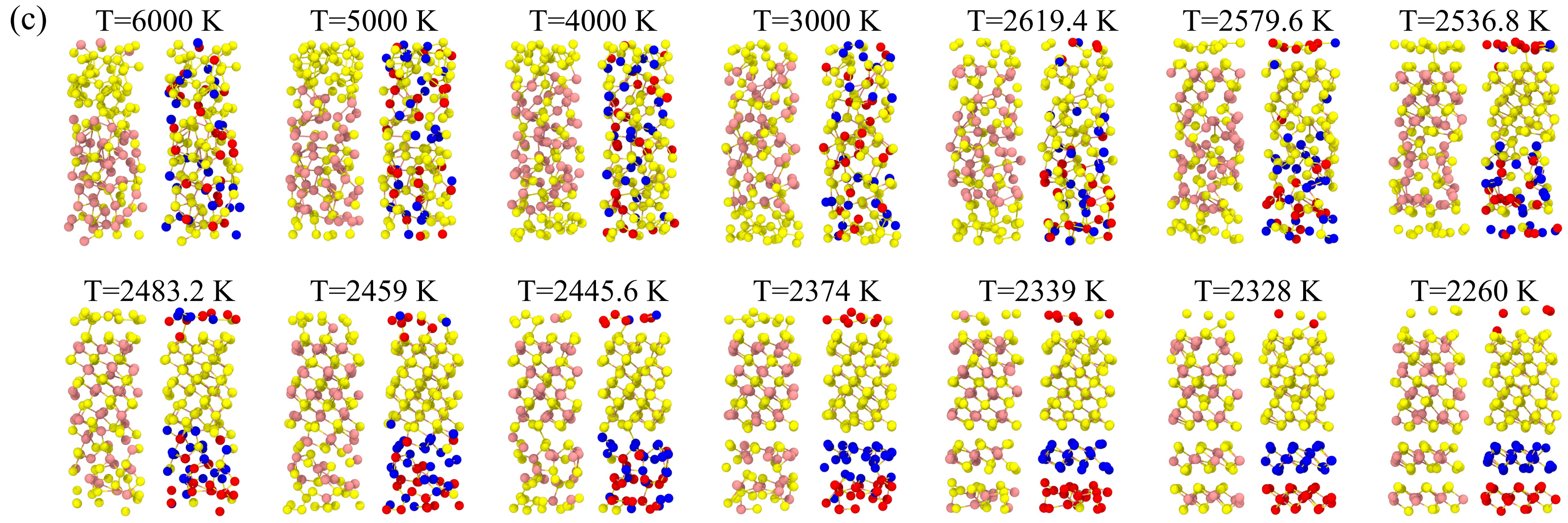

Figure S3. Snapshots of the melt-quench recrystallization trajectories at (a) 6000 K, (b) 5000 K, and (c) 4000 K, showing the time evolution of van der Waals (vdW) layer formation. In each snapshot, the left panel shows atomic configurations colored by element (Bi: light red, Se: yellow), while the right panel highlights atoms based on their vdW layer assignment, with different colors indicating different layers.

<u>ReaxFF reactive force field parameters for Bi/Se</u>

```
Reactive MD-force field: vanadium/bismuth/titanium/molybdenum
 39       ! Number of general parameters
   50.0000 !Overcoordination parameter
    9.5469 !Overcoordination parameter
   26.5405 !Valency angle conjugation parameter
    3.0000 !Triple bond stabilisation parameter
    6.5000 !Triple bond stabilisation parameter
    0.0000 !C2-correction
    1.0588 !Undercoordination parameter
    9.0000 !Triple bond stabilisation parameter
   12.1176 !Undercoordination parameter
   13.3056 !Undercoordination parameter
  -10.0000 !Triple bond stabilization energy
    0.0000 !Lower Taper-radius
   10.0000 !Upper Taper-radius
    2.8793 !Not used
   33.8667 !Valency undercoordination
    6.0891 !Valency angle/lone pair parameter
    1.0563 !Valency angle
    2.0384 !Valency angle parameter
    6.1431 !Not used
    6.9290 !Double bond/angle parameter
    0.3989 !Double bond/angle parameter: overcoord
    3.9954 !Double bond/angle parameter: overcoord
   -2.4837 !Not used
    5.7796 !Torsion/BO parameter
   10.0000 !Torsion overcoordination
    1.9487 !Torsion overcoordination
   -1.2327 !Conjugation 0 (not used)
    2.1645 !Conjugation
    1.5591 !vdWaals shielding
    0.1000 !Cutoff for bond order (*100)
    2.1365 !Valency angle conjugation parameter
    0.6991 !Overcoordination parameter
   50.0000 !Overcoordination parameter
    1.8512 !Valency/lone pair parameter
    0.5000 !Not used
   20.0000 !Not used
    5.0000 !Molecular energy (not used)
    0.0000 !Molecular energy (not used)
    2.6962 !Valency angle conjugation parameter
  2    ! Nr of atoms; cov.r; valency;a.m;Rvdw;Evdw;gammaEEM;cov.r2;#
           alfa;gammavdW;valency;Eunder;Eover;chiEEM;etaEEM;n.u.
           cov r3;Elp;Heat inc.;n.u.;n.u.;n.u.;n.u.
           ov/un;val1;n.u.;val3,vval4
 Bi   2.1949   3.0000 208.9804   2.4429   0.1607   0.4960   0.0535   5.0000
     12.9571  35.5167   3.0000   0.0000   0.0000  -0.1926   6.4153   0.0000
```

```
    -1.0000   0.5785  52.6300   3.8978   0.9856   0.0314   0.8563   0.0000
    -2.5000   5.0597   1.0338   6.0000   2.5791   0.0000   0.0000   0.0000
Se   1.8998   2.0000  78.9710   1.9500   0.5822   1.0000   1.7989   6.0000
     9.0473   2.5977   4.0000  39.6241 112.1416   6.5173   6.0000   2.0000
     1.0000   3.4994  65.0000  16.0645   2.7406   0.7199   0.9745   0.0000
   -25.4997   2.5727   1.0338   6.2998   2.8793   0.0000   0.0000   0.0000
3       ! Nr of bonds; Edis1;LPpen;n.u.;pbe1;pbo5;13corr;pbo6
                        pbe2;pbo3;pbo4;n.u.;pbo1;pbo2;ovcorr
 1  1  66.0677   0.0000   0.0000  -0.9557  -0.2000   0.0000  16.0000  0.2865
        0.5847  -0.2000  15.0000   1.0000  -0.0856   5.2857   0.0000  0.0000
 2  2  60.3243  42.2516   0.0000   1.0000  -0.4781   1.0000  17.8574  0.2125
        0.2308  -0.0681  10.1293   1.0000  -0.1799   4.5000   1.0000  0.0000
 1  2 104.9799  67.4671   0.0000  -0.7758  -0.3000   1.0000  36.0000  0.0100
        4.3525  -0.2016  16.0185   1.0000  -0.1984   3.1634   1.0000  0.0000
 1     ! Nr of off-diagonal terms; Ediss;Ro;gamma;rsigma;rpi;rpi2
 1  2   0.1189   2.0136  13.0000   2.0518   1.5896  -1.0000
5      ! Nr of angles;at1;at2;at3;Thetao,o;ka;kb;pv1;pv2
 2  2  2  83.3840  14.3247   9.1823   0.0000   1.0000   0.0000   1.7839
 1  1  2  65.2859   2.6520   0.7184   0.0000   0.2304   0.0000   1.0760
 2  1  2  78.9382  14.7808   2.2206   0.0000   0.7799   0.0000   3.0000
 1  2  1  90.0000  22.2389   2.9323   0.0000   2.0000   0.0000   1.4182
 1  2  2  34.2463  25.3158   3.0000   0.0000   0.3941   0.0000   1.1866
1      ! Nr of torsions;at1;at2;at3;at4;;V1;V2;V3;V2(BO);vconj;n.u;n
 2  2  2  2   0.0000   0.9033   0.4501  -7.8630   0.0000   0.0000   0.0000
 0     ! Nr of hydrogen bonds;at1;at2;at3;Rhb;Dehb;vhb1
```
